# Dental caries and associated factors in Ethiopia: systematic review and meta-analysis

**DOI:** 10.1186/s12199-021-00943-3

**Published:** 2021-02-12

**Authors:** Tesfu Zewdu, Duresa Abu, Mulatu Agajie, Tamiru Sahilu

**Affiliations:** 1 Department of Nursing, College of Health Science, Assosa University, Assosa, Ethiopia; 2 Department of Pharmacy, College of Health Science, Assosa University, Assosa, Ethiopia

**Keywords:** Dental caries, Meta-analysis, Ethiopia

## Abstract

**Background:**

Globally, dental caries may be a major public health issue which may be preventable. Many studies have been conducted on dental caries in Ethiopia which present inconsistent results.

**Objective:**

This meta-analysis was expected to consolidate the findings conducted in various regions of the country and generate country representative information on the burden and its associated factors of dental caries in Ethiopia.

**Methods:**

Our systematic review and meta-analysis was carried out to estimate the pooled prevalence of dental caries and its associated factors in the Federal Democratic Republic of Ethiopia. Medical specialty databases like ScienceDirect, HINARI, Embase, PubMed, Google Scholar, and Cochrane Library were consistently and exhaustively searched. To determine the aggregate prevalence, studies delineating the prevalence of dental caries and associated factors were included. Important data were extracted employing a standardized data extraction tool ready in Microsoft Excel and imported to the STATA version-13 statistical software package for analyses. To assess non-uniformity, the Cochrane Q test statistics and *I*^2^ test were performed respectively. A random effects model meta-analysis was accustomed to estimate the pooled burden of dental caries.

**Results:**

The result of thirteen studies disclosed that the overall prevalence of dental caries in the Federal Democratic Republic of Ethiopia was found to be 40.98 (31.62, 50.34). Within the subgroup analysis, the uppermost prevalence was determined in Tigray region (46.59% (24.64, 68.54)) whereas the bottom prevalence was determined in Addis Ababa (34.20% (8.42, 59.97)). Dental caries prevalence was considerably high among study subjects who consumed sweet food (OR= 2.4 (95% CI (1.91, 3.01))). But the presence of dental plaque (OR = 5.14 (95% CI (0.67, 39.39))) and habit of tooth- cleaning (OR = 0.71 (95% CI (0.17, 2.96))) were not statistically significant with the outcome of interest.

**Conclusion:**

Our meta-analysis found that the prevalence of dental caries was comparatively high, and sweet food consumption was the most risk issue for dental caries in Ethiopia. The Ethiopian Federal Ministry of Health ought to offer a lot of attention to strengthen the oral health care system and also the implementation of community-level interference programs.

## Background

Dental caries is a bacterial disease that affects the hard tissue of the tooth which is formed through a complex reaction overtime between acid-producing microorganisms and fermentable carbohydrate proceeding to the formation of cavity [1].

Dental caries is a global public health issue worldwide and the most common non-communicable disease. And also most popular condition enclosed in the 2015 Global Burden of Disease study, ranking the 1st for caries of permanent teeth affecting 2.3 billion people and 12th for deciduous teeth affecting 560 million children. The problem is more expensive to treat consuming 5–10% of healthcare budgets in developed countries and common reason for hospitalization. The occurrences of dental caries are increasing because of unlimited use of sugary foods, poor oral care practices, and inadequate health service utilization. The burden of caries was higher in economically disadvantageous community [2–5].

Across all age groups, dental caries affects the quality of life, affected individual and society economy which leads to eating problem, teeth loss and pain, slow language development in children, and absenteeism at school or work [6, 7].

Africa is a home to over 892 million people, and 47 countries carry a special burden of oral health problem and risk factors, approximately 400 million people suffered from oral disease in the region in 2017 according to the WHO report [8]. Many studies revealed that the magnitude of dental caries vary in different countries like Northwest of Spain (36.9%), Nigeria (13.9%), Kenya (50.3%), and Eritrea (78%) respectively [9–12]. Studies that have been conducted in Ethiopia revealed that dental caries accounts for about 21.1–78.2% [13–25]. Although scholars recommended modification of free sugar consumption, cleaning teeth more than two times a day using a fluoride toothpaste (promotion of good oral hygiene), and oral health education, the problem remains high, particularly in developing countries [26, 27].

Even though many studies have been done in Ethiopia on dental caries among different study subjects, the results of them were variable, inconclusive, and inconsistent. Therefore, this review and meta-analysis is expected to describe the aggregate prevalence of dental caries and associated factors in the Federal Democratic Republic of Ethiopia to address the dearth of nationally representative data.

So, better understanding of the aggregate prevalence and contributing factors of dental caries will help the stakeholders to take proper action to control the problem.

## Methods

### Sources of data and ways of search

Studies delineating the burden of dental caries in Ethiopia were consistently and exhaustively searched using medical specialty databases of ScienceDirect, PubMed, Google scholar, Embase, HINARI, and Cochrane Library based on the Preferred Reporting Items for Systematic Reviews and Meta-analysis (PRISMA) guideline [28]. This study was not preregistered. Published and unpublished data (grey pieces of literatures) available on the local university shelves with epidemiological data of dental caries and associated factors done in the Federal Democratic Republic of Ethiopia from 2000 up to 30 September 2020 were incorporated into the review. Citations known by our search terms were taken to EndNote–X7, and duplicate articles were removed. The complete texts of chosen articles were retrieved and read meticulously to determine the quality of the paper. The EndNote software statistical package was employed to cite and download articles. Studies were searched by exploiting the next keywords one by one or in combination: “prevalence of dental caries”, “magnitude of dental caries”, “epidemiology of tooth decay”, “Ethiopia”, “factors related to dental caries”. The search terms were used individually and along with exploitation Boolean operators like “OR” or “AND”.

### Inclusion and exclusion criteria

We included studies delineating the burden of dental caries and associated factors no matter the measurement tool employed; publication type: journal articles; study subjects: all peoples, irrespective of their occupation and sex as well as articles revealed online in the English language were considered; and place of study: solely carried out in Ethiopia from 2000 to 30 September 2020 was included. Articles with methodological issues, incomplete data, and complete text not accessible were excluded from the analysis.

### Information extraction and quality assessment of included studies

The extraction of information (data) was performed by four researchers separately by employing a format ready in Microsoft Excel spreadsheet. In the Microsoft Excel spreadsheet, the following information were incorporated: name of author, publication year, area/region, employed study design, age range, actual sample size, response rate and number of study subjects with the outcome of interest, prevalence rates as well as associated factors considerably related to dental caries. Those articles identified by abstracts and titles were closely reviewed to get back studies with the burden of dental caries. Relevant articles by titles and abstracts were selected for complete text review for its quality and inclusion. Quality of eligible studies was check by employing Newcastle-Ottawa Scale before analysis [29]. Prime quality articles were selected if the scale score was more than half out of 10. Four researchers performed the selection and quality of incorporated articles individually. Discrepancy among the researchers have been resolved through discussion, and articles were included once accord.

### Measurement of outcome of interest

The outcome of interest was dental caries dichotomized as present or absent after rigorous oral examination. Physical examination was performed by dental health professionals by employing necessary instruments. The prevalence was found by dividing the total number of study subjects with the outcome of interest by the total number of study subjects involved in the study multiplied by 100%.

The 2nd outcome of interest in this meta-analysis was factors associated with dental caries closely revealed in three or more studies. The adjusted odds ratio of contributing factors were taken seriously from included studies to determine the most related factor with dental caries in meta-analysis.

### Statistical analysis

Extracted data from primary study by employing a format ready in Microsoft Excel spreadsheet were imported to the STATA version-13 statistical software package for meta-analysis. A meta-analysis of dental caries was performed using random-effects (DerSimonian and Laird) method to adjust for the determined variability [30, 31]. In studies that did not delineate standard error (SE), a SE was calculated in Microsoft excel. And then, the calculated standard error as well as prevalence of every study was imported into the STATA version 13 software to calculate the pooled prevalence rate with 95% CI.

Publication bias was assessed by using a funnel plot through visual assessment. Asymmetry of the funnel plot showed the existence of potential publication bias [32]. Egger’s and Begg’s tests at 5% significant level were also performed; distribution of every study and a *P*-value of less than 0.05 were employed to announce clear existence of publication bias [33]. The non-uniformity among studies was assessed employing Cochran’s Q test (*P*-value less than 0.1 revealed as there was statistically considerable heterogeneity) and inverse variance (*I*^2^) test statistics (which was used to quantify the percentage of total variation in the study estimate because of non-uniformity).

*I*^2^ worth ranges from 0 to 100%. *I*^2^ ≥ 75% indicates high heterogeneousness across studies, and *P*-value < 0.05 was used to announce the presence of a statistically significant heterogeneity [34, 35]. Pooled effect size was calculated, and subgroup analysis was done based on geographical setting (regions) to reduce the random variations between the point effects of the original study. To know the source of non-uniformity, meta-regression was done. Furthermore, point prevalence with 95% CI was displayed on forest plot. On forest plot, the dimension of every box shows the weight of the study. To know the possible associated factors, overall effect was connected in the form of adjusted odds ratio. To identify the effect of every study on the pooled effect size, sensitivity analysis was performed. Four investigators individually done the statistical analysis, and the results were crosschecked for consistency. The included studies’ risk of bias was assessed by employing a 10-item rating scale prepared by Hoy et al. for burden studies [36].

Information collection, sampling, reliability, validity of measurement tool, definition of case, and burden periods of studies were checked. Investigators classified every study as having low risk of bias or “yes” answers to domain questions, high risk of bias or “no” answers to domain questions. Every study was allotted a score of 1 (yes) or 0 (no) for every domain, and these domain scores were summed up to supply the total study quality score. Scores of 8–10 were thought of as having a “low risk of bias,” 6–7 a “moderate risk,” and 0–5 a “high risk”. For the ultimate risk of bias categorization, differences among the reviewers were resolved through agreement.

## Result

### Description of considered studies

**Fig. 1:**
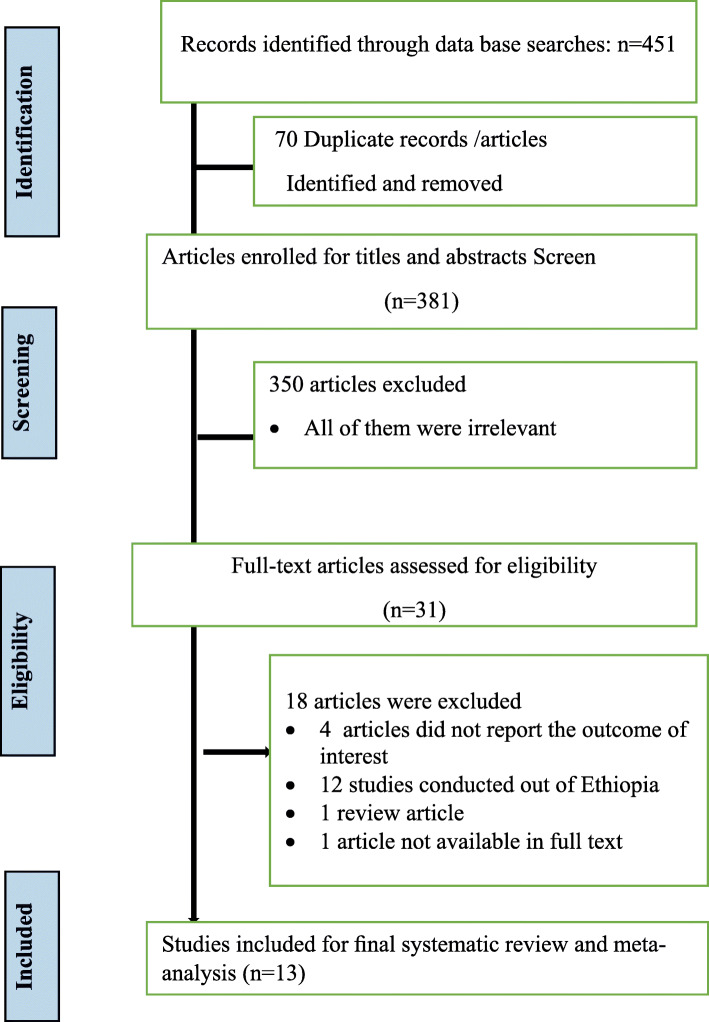
PIRSMA flowchart diagram of selected studies for meta-analysis, Ethiopia

Electronic medical specialty database search engine produced a cumulative of 451 records; from these, 70 duplicate records were identified and rejected. Title and abstract selection triggers rejection of 350 unwanted articles. Then, 31 records underwent complete text review. From 31 records, 18 articles were excluded based on the eligibility criteria; from these, one article was not accessible in full text [9–12, 37–50]. Lastly, 13 articles were incorporated in the final meta-analysis; two of them were pre-prints (Fig. 1). These thirteen studies had a cumulative of 6950 study subjects which were considered in this meta-analysis to determine the overall prevalence of dental caries. Of those, two [20, 25] were conducted in Addis Ababa, six [16–19, 21, 23] in Amhara region, two [14, 24] in Tigray region, one [15] in Oromia region, one [13] in Southern Nations and Nationalities of Ethiopia (SNNPR) region, and one [22] in Harar town. Cross-sectional study design was employed in every study, and studies were conducted among different age groups (their age range 6–100 years) (Table 1). A large proportion of studies were conducted in Amhara region. Ten studies had a sample size above three hundred. The least reported response rate was 82% [18]. The minimum and maximum sample size was 147 and 1736 respectively [18, 25]. All studies were carried out between 2000 and 2020.

**Table 1:** Brief summary of incorporated studies assessing the prevalence of dental caries in Ethiopia, 2020

Author name	Publication year	Region	Study design	Age of subjects	Response rate (%)	Sample size	Total No. of outcome	Prevalence (%)	Quality score
Sehdev et al. [14]	2020	Tigray	Cross-sectional	15–20 years	100%	384	222	57.8	6
Ayele et al. [16]	2013	Amhara	Cross-sectional	7–14 years	100%	842	306	36.3	7
Catherine Simon et al. [25]	2000	Addis Ababa	Cross-sectional	12 years and above	100%	1736	367	21.1	6
Teshome et al. [17]	2020	Amhara	Cross-sectional	6 years and above	100%	368	87	23.64	7
Mulu et al. [18]	2014	Amhara	Cross-sectional	6–15 years	82%	147	32	21.8	8
Aynalem et al. (preprint) [19]	2019	Amhara	Cross-sectional	7–19 years	95%	396	135	34.1	6
Berhane et al. [20]	2014	Addis Ababa	Cross-sectional	10–14 years	100.00%	658	312	47.4	7
Teshome et al. [21]	2016	Amhara	Cross sectional	12–20 years	100.00%	291	141	48.5	8
Ademe et al. [22]	2020	Harar	Cross-sectional	6–15 years	98%	407	150	36.9	8
Tafere et al. [23]	2018	Amhara	Cross-sectional	18 years and above	97.20%	280	219	78.2	8
Zeru et al. [24]	2019	Tigray	Cross-sectional	6–15 years	100.00%	393	139	35.4	7
Komicha et al. [15]	2018	Oromia	Cross-sectional	20 years and above	100.00%	422	133	31.5	7
Bogale et al. (preprints) [13]	2019	SNNPR	Cross-sectional	18–100 years	89.30%	626	377	60.2	7

### Meta-analysis

**Fig. 2:**
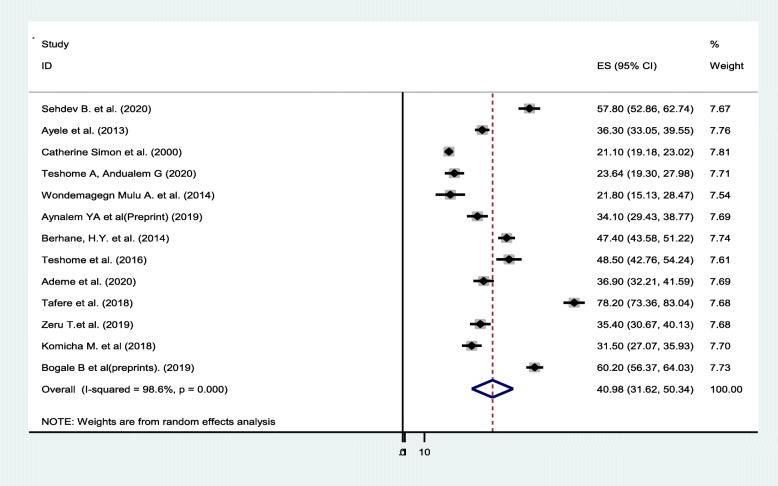
Forest plot revealing overall prevalence of dental caries in Ethiopia

**Fig. 3:**
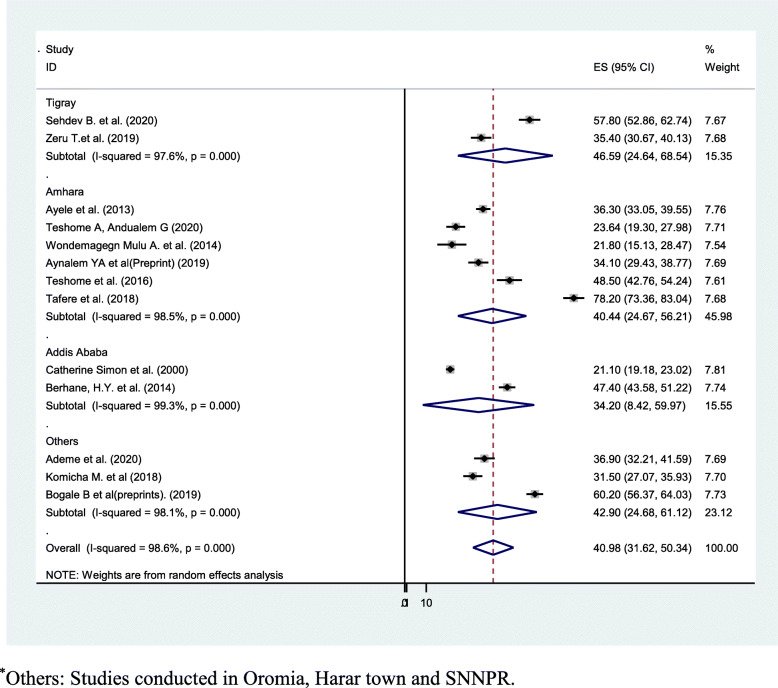
Forest plot showing the subgroup analysis of dental caries in Ethiopia by region. *Others: studies conducted in Oromia, Harar town, and SNNPR

**Fig. 4:**
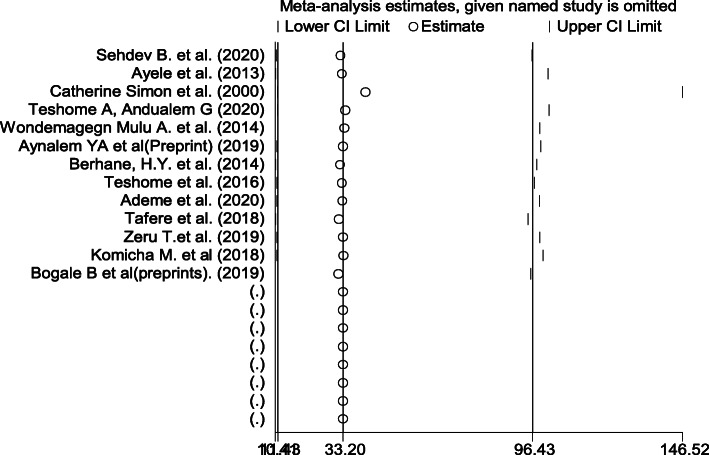
Output of sensitivity analysis of 13 studies

**Fig. 5:**
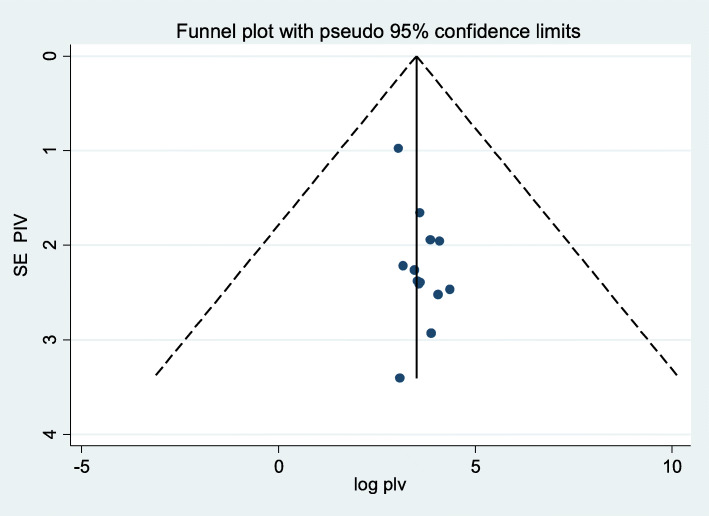
Funnel plot test for publication bias, Ethiopia

Overall pooled prevalence of dental caries was found to be 40.98 (31.62, 50.34). The test statistic showed high non-uniformity among every study (*I*^2^ = 98.6%, *P* = 0.000) (Fig. 2). Due to this reason, random effects model was accustomed to estimate the DerSimonian and Laird overall effect. Furthermore, during this meta-analysis, subgroup analysis was strictly performed depending on the region of the studies conducted. The highest pooled prevalence of dental caries was determined in Tigray region of Ethiopia (46.59% (24.64, 68.54)) and the lowest prevalence of dental caries was determined in Addis Ababa (34.20% (8.42, 59.97)) (Fig. 3). On the subgroup analysis, the output still revealed as there was heterogeneity across studies. We carried out meta-regression analysis employing sample size and publication year as a covariate (Table 2). None of these variables were statistically considerable source of non-uniformity. Also, sensitivity analysis was carried out to identify the effect of every study on the overall effect size. The result of this analysis did not show a single study that considerably influenced the cumulative pooled prevalence of dental caries (Fig. 4). We have checked publication bias by looking at the funnel plot for its symmetry (Fig. 5).

**Table 2:** Meta-regression analysis of factors that have influence between study non-uniformity

Covariate	Coefficients	Std. Err	*P*-value
Publication year	1.611	1.912	0.419
Sample size	0.004	0.022	0.864

The funnel plot revealed that there was symmetrical distribution of incorporated studies. Begg’s test showed absence of publication bias (Pr > |z| = 0.393).

**Fig. 6:**
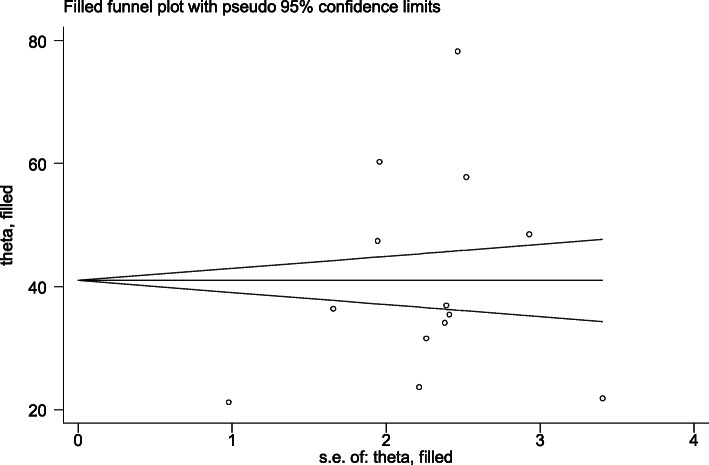
Trim and fill analysis for correcting publication bias of 13 studies

However, the output of Egger’s test was statistically considerable for the existence of publication bias (*P* = 0.030). And also, we have done trim and fill analysis (Fig. 6). After meta-trim and fill analysis, the pooled value is 40.984 with 95% CI (31.623, 50.344). There is no significant change because the confidence intervals of the two findings (before and after meta-trim) overlapped.

### Associated factors of caries

**Fig. 7:**
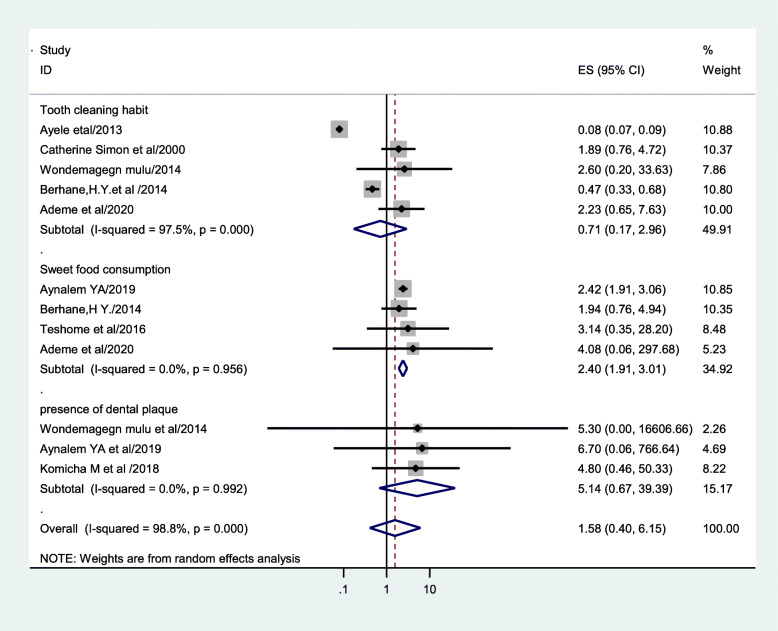
Forest plot revealing associate factors of dental caries in Ethiopia, 2000–2020

Four studies have been evaluated to check the presence of association between sweet food consumption and caries [19–22]. Dental caries prevalence was considerably high among study subjects who consumed sweet food (OR= 2.4 (95% CI (1.91, 3.01))). The analysis of three studies [15, 18, 19] found that presence of dental plaque was not considerably associated with dental caries (OR = 5.14 (95% CI (0.67, 39.39))) (Fig. 7). The pooled regression analysis of five articles [16, 18, 20, 22, 25] showed no association between habit of tooth cleaning and dental caries (OR =0.71(95 %( CI (0.17, 2.96))). Non-uniformity tests disclosed that there was high non-uniformity in studies that evaluate habit of tooth cleaning (*I*^2^ = 97.5%, *P* = 0.000). No publication bias was detected for all factors as manifested by Egger’s regression test (Egger’s test = 0.074).

## Discussion

Currently, there is an increase in the burden of dental caries in line with emerging economies in developing countries but decreasing in developed nations because of improved oral hygiene and also implementation of community-level interference programs. But an increase in dental caries burden more likely related with lack of good oral health care system because the system mostly focuses on curative care but no periodical implementation of community oral health promotion [51, 52].

Data containing 6950 study subjects from 13 studies were incorporated in this study. The result of this meta-analysis found that the pooled prevalence of dental caries was found to be 40.98% (31.62, 50.34), which is similar with previous studies carried out in the USA (37%) [53], India (36.5%) [54], and Kenya (43.3%) [55]. However, the estimate of dental caries in this meta-analysis was lower than studies done in China (74.7%) [56], India (68.8%) [57], and oral health report of school children by WHO [60–90%] [58], and higher than study conducted in Sudan (30.5%) [59]. The possible reasons for the difference might be due to socio-economic differences or diversity in dietary habits, oral hygiene practices, differences in knowledge and attitude, and implementation of community-level prevention programs among the above stated country’s population [60, 61].

Within subgroup analysis by region, the uppermost prevalence of dental caries was revealed in Tigray region of Ethiopia (46.59% (24.64, 68.54)), and the bottom prevalence of dental caries was detected in Addis Ababa (34.20% (8.42, 59.97)). This might be due to difference in sample size and poor oral health education in the abovementioned areas as compared to other regions. This meta-analysis also found that sweet food consumption had an influence on dental caries, but the habit of tooth cleaning and the presence of dental plaque did not had an influence.

The consumption of sweet food increases the odds of advancing dental caries by more than double (OR=2.4(95% CI (1.91, 3.01))), which is comparable with a study done in Kenya and Brazil [62, 63]. The finding on sweet food consumption is somewhat expected because the combination of different factors like the colonization of cariogenic bacteria on teeth, sugar consumption very often, and sensitive teeth increases the risk of developing tooth decay. Sucrose is the most cariogenic sugar because it will type glucan, a substance that permits bacteria to stick higher to the teeth, or high consumption of drinks that are sweetened with sugar is the most risk factor for developing caries [64]. The presence of dental plaque did not have much estimate of positive effect on the odds of developing dental caries (OR= 5.14(95 %CI (0.67, 39.39))). This result was incomparable with previous studies conducted in Mexico and Belgium [65, 66]. The habit of tooth cleaning had no significant association with the development of dental caries in our study (OR = 0.71(95 % CI (0.17, 2.96))). This non-considerable association might be because of few numbers of studies employed to estimate pooled effect size.

Even if this meta-analysis provided important data and recent evidence of prevalence of dental caries in Ethiopia, there were some limitations. First, we only scrutinized the effect of three factors because other main determinant factors were not systematically investigated across incorporated studies, and second, the search terms were restricted to articles published in English

## Conclusion and recommendations

This meta-analysis found that the prevalence of dental caries in Ethiopia was comparatively high. Dental caries among study population was significantly associated with sweet food consumption very often but not with the presence of dental plaque and habit of tooth cleaning. Our result suggests that Ethiopian Federal Ministry of Health ought to offer a lot of attention to strengthen the oral health care system and also the implementation of community-level interference programs.

In the future, further research is expected to know the detail of contributing factors of dental caries for appropriate intervention or explore context-specifics strategies.

## Data Availability

All needed data are available from corresponding author upon rational request.

## Abbreviations

CI: Confidence interval
*I*^2^: Inverse variance
OR: Odds ratio
PRISMA: Preferred Reporting Items for Systematic Reviews and Meta-analysis
SE: Standard error
SNNPR: Southern Nations and Nationalities of Ethiopia
WHO: World Health Organization
